# The Oration Delivered before the Medical Society of London, at Their Sixty-Eighth Anniversary, March 8, 1841

**Published:** 1842-01

**Authors:** 


Art. VI.— *The Oration delivered before the Medical Society of London, at their Sixty-eighth Anniversary, March* 8, 1841. By W. D. Chowne, M.D., &c. Printed at the request of the Society.—*London,* 1841. 8vo, pp. 71.

We have been both interested and instructed by the perusal of this performance. It exhibits and illustrates in a lucid manner the means by which medical science is *advanced,* and the uses to which it is *applied.* The whole manner of the oration evinces a degree of candour, learning, and humanity, alike honorable to the author and the society at whose sixty-eighth anniversary it was delivered.

